# Supplementary material for: Letermovir Rescue Therapy in Kidney Transplant Recipients with Refractory/Resistant CMV Disease
Source: J Clin Med. 2023 Dec 23;13(1):100. doi: 10.3390/jcm13010100 (PMC10780128; doi:10.3390/jcm13010100)
Supplement: Supplementary file 1 [file jcm-13-00100-s001.zip › Table S1.pdf]

Case 1

CMV-DNA	cop/ml	Leukocytes	/nl	Lymphocytes	/nl	Kreatinin	mg/dl	eGFR	ml/min	ALT	U/l	GGT	U/l	Bilirubin	mg/dl
17.10.22	negativ	17.10.22	7.06	19.07.22	2.25	17.10.22	1.94	17.10.22	44	23.05.22	38	23.05.22	30	07.01.22	0.61
30.08.22	negativ	05.09.22	6.65	07.01.22	1.26	05.09.22	1.7	05.09.22	52	28.04.22	41	28.04.22	27	12.11.21	0.47
19.07.22	negativ	30.08.22	7.08	12.11.21	0.59	30.08.22	1.7	30.08.22	52	22.02.22	39	22.02.22	28	15.06.21	0.29
29.06.22	negativ	19.07.22	8.76	30.10.20	0.99	19.07.22	1.77	19.07.22	50	03.02.22	40	03.02.22	26	18.05.21	0.28
15.06.22	<2000	29.06.22	7.64			29.06.22	1.98	29.06.22	43	07.01.22	43	07.01.22	34	20.04.21	0.34
23.05.22	<2000	29.06.22	8.03			15.06.22	1.64	15.06.22	55	02.12.21	97	02.12.21	31	23.03.21	0.40
28.04.22	negativ	15.06.22	5.19			15.03.22	1.58	15.03.22	57	24.11.21	129	24.11.21	33	30.10.20	0.20
15.03.22	<2000	15.03.22	7.73			01.03.22	1.64	01.03.22	55	12.11.21	82	12.11.21	26	06.10.20	0.18
22.02.22	<2000	01.03.22	8.49			22.02.22	1.52	22.02.22	60	04.11.21	60	04.11.21	32	28.09.20	0.21
03.02.22	<2000	22.02.22	7.14			14.02.22	1.58	14.02.22	57	18.05.21	38	18.05.21	24	21.09.20	0.15
07.01.22	<2000	14.02.22	7.44			03.02.22	1.6	03.02.22	56	20.04.21	35	20.04.21	24	17.09.20	0.18
09.12.21	<2000	03.02.22	8.61			10.01.22	1.69	10.01.22	53	23.03.21	63	30.10.20	16	16.09.20	0.24
06.12.21	<2000	10.01.22	10.5			07.01.22	2.13	07.01.22	40	30.10.20	26	06.10.20	21	15.09.20	0.69
24.11.21	13300	07.01.22	8.43			09.12.21	1.51	09.12.21	60					14.09.20	0.34
17.11.21	7960	09.12.21	5.86			02.12.21	1.65	02.12.21	54					08.07.20	0.21
12.11.21	<2000	02.12.21	5.26			24.11.21	1.69	24.11.21	53						
11.11.21	6940	24.11.21	6.12			17.11.21	1.8	17.11.21	49						
04.11.21	10200	17.11.21	5.07			12.11.21	1.62	12.11.21	55						
08.10.21	<2000	12.11.21	5.14			04.11.21	1.4	04.11.21	66						
09.09.21	<2000	04.11.21	5.09			08.10.21	1.72	08.10.21	52						
10.08.21	<2000	08.10.21	6.14			09.09.21	1.72	09.09.21	52						
13.07.21	<2000	09.09.21	5.74			10.08.21	1.64	10.08.21	55						
15.06.21	<2000	10.08.21	5.05			13.07.21	1.87	13.07.21	47						
18.05.21	<2000	13.07.21	4.73			15.06.21	1.65	15.06.21	54						
20.04.21	<2000	15.06.21	4.96			18.05.21	1.85	18.05.21	47						

23.03.21	38900	18.05.21	4.66			20.04.21	1.95	20.04.21	45						
23.02.21	<2000	20.04.21	5.96			23.03.21	2.21	23.03.21	38						
26.01.21	negativ	23.03.21	3.91			23.03.21	2.33	23.03.21	36						
29.12.20	negativ	23.02.21	4.08			23.02.21	2.19	23.02.21	39						
		26.01.21	3.98			26.01.21	2.21	26.01.21	38						
		29.12.20	3.77			29.12.20	2.07	29.12.20	41						

Case 2

CMV-DNA	cop/ml	Leukocytes	/nl	Lymphocytes	/nl	Kreatinin	mg/dl	eGFR	ml/min	ALT	U/l	GGT	U/l	Bilirubin	mg/dl
13.04.23	<2000	13.04.2023	13.60	13.04.2023	3.51	13.04.2023	2.03	13.04.2023	23	13.04.23	14	13.04.23	22	13.04.23	0.41
08.09.22	<2000	13.12.2022	10.44	09.06.2022	3.53	13.12.2022	2.19	13.12.2022	21	13.12.22	10	13.12.22	21	08.09.22	0.50
17.03.22	<2000	08.09.2022	10.57	17.03.2022	3.86	08.09.2022	2.15	08.09.2022	22	08.09.22	18	08.09.22	22	17.03.22	0.58
14.12.21	negativ	09.06.2022	12.40	14.12.2021	3.64	09.06.2022	2.12	09.06.2022	22	09.06.22	18	09.06.22	25	28.10.21	0.40
28.10.21	<2000	17.03.2022	10.96	28.10.2021	3.52	17.03.2022	1.97	17.03.2022	25	17.03.22	40	17.03.22	44	29.04.21	0.32
29.04.21	<2000	14.12.2021	10.34	03.06.2021	2.87	14.12.2021	2.10	14.12.2021	23	14.12.21	23	14.12.21	24	15.03.21	0.16
15.03.21	negativ	28.10.2021	11.60	29.04.2021	2.64	28.10.2021	2.39	28.10.2021	19	28.10.21	16	28.10.21	25	01.03.21	0.15
24.02.21	<2000	19.08.2021	11.07	15.03.2021	3.80	19.08.2021	1.91	19.08.2021	26	19.08.21	23	19.08.21	41	24.02.21	0.15
12.01.21	<2000	03.06.2021	8.48	01.03.2021	1.95	03.06.2021	2.18	03.06.2021	22	03.06.21	21	03.06.21	25	18.02.21	0.18
12.11.20	<2000	29.04.2021	10.92	24.02.2021	2.86	29.04.2021	1.99	29.04.2021	24	29.04.21	20	29.04.21	41	16.02.21	<0.15
15.10.20	<2000	15.03.2021	9.19	18.02.2021	2.93	15.03.2021	2.64	15.03.2021	17	15.03.21	16	15.03.21	47	13.02.21	<0.15
31.08.20	<2000	07.03.2021	6.73	16.02.2021	2.57	07.03.2021	2.19	07.03.2021	22	01.03.21	15	01.03.21	38	03.02.21	<0.14
10.08.20	<2000	05.03.2021	7.66	13.02.2021	2.03	05.03.2021	2.55	05.03.2021	18	24.02.21	25	24.02.21	50	02.02.21	0.15
18.06.20	<2000	01.03.2021	7.99	02.02.2021	1.48	01.03.2021	2.97	01.03.2021	15	18.02.21	22	18.02.21	49	01.02.21	<0.15
19.05.20	<2000	24.02.2021	8.86	01.02.2021	1.84	24.02.2021	3.36	24.02.2021	<15	16.02.21	23	16.02.21	46	31.01.21	<0.15
05.05.20	18500.00	18.02.2021	10.28	15.10.2020	1.79	18.02.2021	3.62	18.02.2021	<15	13.02.21	18	13.02.21	38	30.01.21	<0.15
22.04.20	187000.00	16.02.2021	10.14	28.07.2020	1.30	16.02.2021	4.07	16.02.2021	<15	04.02.21	6	04.02.21	35	28.01.21	<0.15

14.04.20	4360000.00	13.02.2021	8.61	29.05.2020	1.34	13.02.2021	4.68	13.02.2021	<15	03.02.21	8	03.02.21	37	26.01.21	<0.15
01.04.20	692000.00	11.02.2021	5.14	03.01.2020	0.46	11.02.2021	5.35	11.02.2021	<15	02.02.21	7	02.02.21	38	25.01.21	0.16
19.03.20	202000.00	09.02.2021	7.50	28.10.2019	0.82	09.02.2021	5.66	09.02.2021	<15	01.02.21	10	01.02.21	52	24.01.21	<0.15
27.02.20	57100.00	08.02.2021	7.32	14.10.2019	0.77	08.02.2021	5.67	08.02.2021	<15	31.01.21	8	31.01.21	43	22.01.21	0.22
18.02.20	43000.00	07.02.2021	6.84			07.02.2021	5.57	07.02.2021	<15	28.01.21	10	28.01.21	46	20.01.21	0.32
11.02.20	46600.00	05.02.2021	6.47			05.02.2021	4.67	05.02.2021	<15	26.01.21	7	26.01.21	53	18.01.21	0.21
04.02.20	20400.00	04.02.2021	6.99			04.02.2021	4.35	04.02.2021	<15	25.01.21	<5	25.01.21	45	16.01.21	0.32
03.01.20	<2000	03.02.2021	5.74			03.02.2021	3.69	03.02.2021	<15	24.01.21	6	24.01.21	41	14.01.21	0.29
27.12.19	<2000	02.02.2021	6.71			02.02.2021	3.45	02.02.2021	<15	22.01.21	<5	22.01.21	34	13.01.21	0.23
27.11.19	negativ	01.02.2021	6.79			01.02.2021	3.28	01.02.2021	<15	20.01.21	6	20.01.21	46	12.01.21	0.27
07.11.19	negativ	31.01.2021	6.41			31.01.2021	3.59	31.01.2021	<15	18.01.21	<5	18.01.21	52	15.10.20	0.28
14.10.19	negativ	30.01.2021	5.07			30.01.2021	3.45	30.01.2021	<15	16.01.21	30	16.01.21	60	14.04.20	0.35
		29.01.2021	5.71			29.01.2021	3.35	29.01.2021	<15	14.01.21	18	14.01.21	57	01.04.20	0.36
		28.01.2021	5.07			28.01.2021	2.59	28.01.2021	18	13.01.21	14	13.01.21	33	03.01.20	0.18
		26.01.2021	5.51			26.01.2021	2.11	26.01.2021	23	12.01.21	11	12.01.21	19	27.12.19	0.17
		25.01.2021	5.34			25.01.2021	1.95	25.01.2021	25	10.01.21	15	10.01.21	39	27.11.19	0.22
		24.01.2021	7.67			24.01.2021	2.00	24.01.2021	24	12.11.20	20	12.11.20	31	07.11.19	0.22
		22.01.2021	7.78			22.01.2021	2.08	22.01.2021	23	15.10.20	18	15.10.20	29	14.10.19	0.28
		20.01.2021	9.21			20.01.2021	2.47	20.01.2021	19	30.09.20	24	30.09.20	29		
		18.01.2021	8.95			18.01.2021	2.30	18.01.2021	20	31.08.20	34	31.08.20	63		
		16.01.2021	7.67			16.01.2021	2.51	16.01.2021	18	10.08.20	62	10.08.20	126		
		14.01.2021	10.10			14.01.2021	2.65	14.01.2021	17	23.07.20	26	18.06.20	76		
		13.01.2021	5.29			13.01.2021	2.76	13.01.2021	16	23.07.20	26	19.05.20	74		
		12.01.2021	5.91			12.01.2021	3.49	12.01.2021	<15	18.06.20	30	05.05.20	69		
		10.01.2021	13.33			10.01.2021	3.12	10.01.2021	<15	29.05.20	34	22.04.20	77		
		12.11.2020	9.50			12.11.2020	1.88	12.11.2020	26	19.05.20	47	14.04.20	46		
		15.10.2020	9.83			15.10.2020	1.73	15.10.2020	29	05.05.20	46	01.04.20	55		
		30.09.2020	8.45			30.09.2020	1.94	30.09.2020	25	22.04.20	65	19.03.20	40		

31.08.2020	8.88	31.08.2020	1.67	31.08.2020	30	14.04.20	42	27.02.20	28
10.08.2020	9.71	10.08.2020	1.46	10.08.2020	36	01.04.20	59	18.02.20	31
02.08.2020	8.48	02.08.2020	1.35	02.08.2020	39	19.03.20	43	11.02.20	35
01.08.2020	12.82	01.08.2020	1.50	01.08.2020	35	27.02.20	22	04.02.20	29
28.07.2020	7.43	28.07.2020	1.38	28.07.2020	38	18.02.20	27	03.01.20	20
27.07.2020	8.52	27.07.2020	1.55	27.07.2020	33	11.02.20	33	27.12.19	19
23.07.2020	10.41	23.07.2020	1.44	23.07.2020	36	04.02.20	31	12.12.19	16
18.06.2020	11.50	18.06.2020	1.54	18.06.2020	33	03.01.20	15	27.11.19	19
03.06.2020	9.36	03.06.2020	1.77	03.06.2020	28	27.12.19	11	27.11.19	23
29.05.2020	9.85	29.05.2020	1.63	29.05.2020	31	12.12.19	12	14.11.19	28
19.05.2020	10.62	19.05.2020	1.46	19.05.2020	36	27.11.19	8	07.11.19	36
05.05.2020	10.31	05.05.2020	1.59	05.05.2020	32	27.11.19	10	28.10.19	80
22.04.2020	5.57	22.04.2020	1.31	22.04.2020	41	14.11.19	13	17.10.19	79
14.04.2020	3.71	14.04.2020	1.33	14.04.2020	40	07.11.19	9	14.10.19	53
01.04.2020	2.86	01.04.2020	1.37	01.04.2020	39	28.10.19	22		
19.03.2020	3.20	19.03.2020	1.59	19.03.2020	32	23.10.19	26		
27.02.2020	2.89	27.02.2020	1.48	27.02.2020	35	17.10.19	38		
18.02.2020	2.43	18.02.2020	1.41	18.02.2020	37	14.10.19	33		
11.02.2020	2.84	11.02.2020	1.43	11.02.2020	37				
04.02.2020	3.20	04.02.2020	1.50	04.02.2020	35				
03.01.2020	3.35	03.01.2020	1.48	03.01.2020	35				
27.12.2019	3.18	27.12.2019	1.87	27.12.2019	26				
12.12.2019	5.46	12.12.2019	1.75	12.12.2019	29				
27.11.2019	9.74	27.11.2019	1.72	27.11.2019	28				
27.11.2019	9.89	27.11.2019	1.77	27.11.2019	29				
14.11.2019	8.59	14.11.2019	1.79	14.11.2019	28				
07.11.2019	10.63	07.11.2019	1.97	07.11.2019	25				
28.10.2019	9.53	28.10.2019	1.60	28.10.2019	32				

		25.10.2019	7.11			25.10.2019	1.76	25.10.2019	28						
		23.10.2019	8.12			23.10.2019	1.75	23.10.2019	29						
		17.10.2019	11.52			17.10.2019	1.57	17.10.2019	33						
		14.10.2019	12.62			14.10.2019	1.78	14.10.2019	28						

Case 3

CMV-DNA	cop/ml	Leukocytes	/nl	Lymphocytes	/nl	Kreatinin	mg/dl	eGFR	ml/min	ALT	U/l	GGT	U/l	Bilirubin	mg/dl
03.05.2023	<2000	03.05.2023	5.89	03.05.2023	0.94	03.05.2023	2.42	03.05.2023	29	03.05.23	10	03.05.23	27	03.05.23	0.33
08.02.2023	<2000	08.02.2023	3.94	08.02.2023	0.71	08.02.2023	3.06	08.02.2023	22	08.02.23	9	08.02.23	20	08.02.23	0.64
16.11.2022	<2000	16.11.2022	3.95	23.05.2022	0.70	16.11.2022	2.60	16.11.2022	26	16.11.22	<5	16.11.22	22	23.05.22	0.50
22.08.2022	<2000	22.08.2022	4.76	16.01.2020	1.70	22.08.2022	2.67	22.08.2022	26	22.08.22	10	22.08.22	19	04.01.21	0.47
23.05.2022	schwach positiv	23.05.2022	3.89	27.11.2019	1.13	23.05.2022	2.41	23.05.2022	29	23.05.22	7	23.05.22	20	16.01.20	0.59
25.03.2021	<2000	02.12.2021	4.67	01.10.2019	2.08	02.12.2021	2.60	02.12.2021	27	02.12.21	11	02.12.21	25	27.11.19	0.63
04.01.2021	<2000	09.09.2021	4.34	10.09.2019	1.78	09.09.2021	2.80	09.09.2021	24	09.09.21	9	09.09.21	25	01.10.19	0.54
12.03.2020	negativ	17.06.2021	4.24	29.08.2019	1.80	17.06.2021	2.74	17.06.2021	25	17.06.21	10	17.06.21	26	02.05.19	0.30
13.02.2020	<2000	25.03.2021	4.08	02.05.2019	0.85	25.03.2021	2.77	25.03.2021	25	25.03.21	12	25.03.21	23	02.05.19	0.32
16.01.2020	<2000	08.10.2020	4.81	05.04.2019	0.23	04.01.2021	2.95	04.01.2021	23	04.01.21	13	04.01.21	29	15.04.19	0.69
05.11.2019	<2000	16.07.2020	4.96	07.02.2019	0.59	08.10.2020	3.03	08.10.2020	22	08.10.20	8	08.10.20	31	04.04.19	0.44
01.10.2019	<2000	18.06.2020	5.01	24.01.2019	0.48	16.07.2020	2.71	16.07.2020	26	16.07.20	8	16.07.20	28	07.02.19	0.46
10.09.2019	<2000	12.03.2020	4.30	10.01.2019	0.57	18.06.2020	2.70	18.06.2020	26	18.06.20	10	18.06.20	32	24.01.19	0.42
29.08.2019	<2000	28.02.2020	4.30	28.12.2018	1.01	12.03.2020	3.47	12.03.2020	19	12.03.20	10	12.03.20	34	10.01.19	0.41
01.08.2019	4640	13.02.2020	4.76	21.12.2018	0.90	28.02.2020	3.26	28.02.2020	20	28.02.20	10	28.02.20	36	02.01.19	0.40
03.07.2019	4390	30.01.2020	3.78			13.02.2020	3.36	13.02.2020	20	13.02.20	13	13.02.20	40	28.12.18	0.44
29.05.2019	383000	16.01.2020	4.68			30.01.2020	3.34	30.01.2020	20	30.01.20	13	30.01.20	47	28.12.18	0.42
02.05.2019	229000	11.12.2019	6.61			30.01.2020	3.45	30.01.2020	19	16.01.20	15	16.01.20	51	21.12.18	0.37

17.04.2019	18600000	27.11.2019	5.48	16.01.2020	3.33	16.01.2020	20	11.12.19	14	11.12.19	41
15.04.2019	7590000	05.11.2019	5.14	11.12.2019	3.79	11.12.2019	17	27.11.19	10	27.11.19	34
11.04.2019	4270000	01.10.2019	5.21	27.11.2019	3.64	27.11.2019	18	05.11.19	11	05.11.19	35
07.04.2019	4520000	10.09.2019	5.10	05.11.2019	3.45	05.11.2019	19	01.10.19	13	01.10.19	40
04.04.2019	5120000	29.08.2019	5.64	01.10.2019	3.29	01.10.2019	20	10.09.19	10	10.09.19	41
06.03.2019	33400	03.07.2019	4.17	10.09.2019	3.07	10.09.2019	22	29.08.19	7	29.08.19	45
07.02.2019	<2000	29.05.2019	3.74	29.08.2019	3.95	29.08.2019	16	01.08.19	6	01.08.19	43
24.01.2019	<2000	02.05.2019	4.94	01.08.2019	3.55	03.07.2019	20	03.07.19	7	03.07.19	40
10.01.2019	2940	02.05.2019	4.90	03.07.2019	3.41	29.05.2019	22	29.05.19	8	29.05.19	47
02.01.2019	<2000	17.04.2019	4.53	29.05.2019	3.09	02.05.2019	26	02.05.19	11	02.05.19	62
28.12.2018	negativ	16.04.2019	5.09	02.05.2019	2.80	02.05.2019	25	02.05.19	7	02.05.19	63
21.12.2018	negativ	15.04.2019	4.87	02.05.2019	2.72	17.04.2019	22	15.04.19	10	15.04.19	69
		12.04.2019	3.13	17.04.2019	3.04	16.04.2019	24	05.04.19	9	05.04.19	47
		11.04.2019	3.10	16.04.2019	2.90	15.04.2019	22	04.04.19	10	04.04.19	57
		10.04.2019	3.24	15.04.2019	3.11	12.04.2019	22	04.04.19	11	04.04.19	57
		09.04.2019	3.18	12.04.2019	3.12	11.04.2019	20	06.03.19	8	06.03.19	22
		07.04.2019	3.08	11.04.2019	3.36	10.04.2019	21	07.02.19	<5 U/l	07.02.19	21
		05.04.2019	3.36	10.04.2019	3.18	09.04.2019	21	24.01.19	6	24.01.19	21
		04.04.2019	2.97	09.04.2019	3.24	07.04.2019	19	10.01.19	5	10.01.19	23
		04.04.2019	2.92	07.04.2019	3.44	05.04.2019	20	02.01.19	5	28.12.18	21
		06.03.2019	2.87	05.04.2019	3.30	04.04.2019	19	28.12.18	6	28.12.18	20
		07.02.2019	3.49	04.04.2019	3.56	04.04.2019	19	28.12.18	6	21.12.18	22
		24.01.2019	2.90	04.04.2019	3.49	06.03.2019	20	21.12.18	6		
		10.01.2019	3.74	06.03.2019	3.41	07.02.2019	21				
		02.01.2019	4.20	07.02.2019	3.18	24.01.2019	18				
		30.12.2018	5.65	24.01.2019	3.62	10.01.2019	<15				
		28.12.2018	4.88	10.01.2019	4.43	02.01.2019	16				
		28.12.2018	4.80	02.01.2019	4.07	30.12.2018	16				

		21.12.2018	4.11			30.12.2018	4.08	28.12.2018	<15						
						28.12.2018	4.61	28.12.2018	<15						
						28.12.2018	4.40	21.12.2018	16						
						21.12.2018	3.99	21.12.2018	16						

Case 4

CMV-DNA	cop/ml	Leukocytes	/nl	Lymphocytes	/nl	Kreatinin	mg/dl	eGFR	ml/min	ALT	U/l	GGT	U/l	Bilirubin	mg/dl
11.04.2023	negativ	11.04.2023	6.92	13.02.2023	2.14	11.04.2023	1.42	11.04.2023	59	11.04.23	36	15.02.22	44	07.02.22	1.00
13.02.2023	<2000	13.02.2023	6.21	07.02.2022	0.91	13.02.2023	1.27	13.02.2023	68	13.02.23	30	07.02.22	47	23.09.21	0.21
15.11.2022	negativ	15.11.2022	7.12	06.09.2021	1.37	15.11.2022	1.34	15.11.2022	64	15.11.22	34	20.12.21	34	16.09.21	0.23
03.08.2022	<2000	03.08.2022	6.01			03.08.2022	1.24	03.08.2022	70	03.08.22	40	29.11.21	35		
30.05.2022	<2000	30.05.2022	6.10			30.05.2022	1.16	30.05.2022	76	30.05.22	53	15.11.21	27		
28.03.2022	4270	28.03.2022	5.38			28.03.2022	1.09	28.03.2022	82	28.03.22	56	19.10.21	30		
15.02.2022	205000	15.02.2022	4.86			15.02.2022	1.25	15.02.2022	70	01.11.21	70	23.09.21	34		
07.02.2022	980000	07.02.2022	3.72			07.02.2022	1.47	07.02.2022	57						
20.12.2021	<2000	20.12.2021	4.54			20.12.2021	1.67	20.12.2021	49						
29.11.2021	<2000	29.11.2021	5.20			29.11.2021	1.87	29.11.2021	43						
15.11.2021	5290	15.11.2021	6.35			15.11.2021	2.48	15.11.2021	30						
01.11.2021	2700	01.11.2021	4.52			01.11.2021	1.99	01.11.2021	40						
19.10.2021	negativ	19.10.2021	6.58			19.10.2021	2.65	19.10.2021	28						
30.09.2021	negativ	14.10.2021	7.42			14.10.2021	2.20	14.10.2021	35						
		12.10.2021	8.30			12.10.2021	2.95	12.10.2021	25						
		30.09.2021	7.76			30.09.2021	2.92	30.09.2021	25						

Case 5

CMV-DNA	cop/ml	Leukocytes	/nl	Lymphocytes	/nl	Kreatinin	mg/dl	eGFR	ml/min	ALT	U/l	GGT	U/l	Bilirubin	mg/dl
05.01.23	<2000	18.01.2023	8.75	05.01.2023	0.82	05.01.2023	3.25	05.01.2023	17	24.10.22	21	24.10.22	80	04.01.23	0.45
24.10.22	negativ	05.01.2023	18.66	04.01.2023	1.16	04.01.2023	3.48	04.01.2023	16	02.03.22	13	02.03.22	21	25.08.22	0.36
25.08.22	negativ	04.01.2023	19.15	25.08.2022	2.29	24.10.2022	3.17	24.10.2022	18	01.12.21	13	01.12.21	17	01.05.22	0.67
10.05.22	12000.00	24.10.2022	8.24	01.05.2022	1.26	25.08.2022	2.94	25.08.2022	19	14.09.21	14	14.09.21	18	21.04.22	0.50
02.05.22	36900.00	25.08.2022	9.00	29.04.2022	1.20	17.05.2022	3.33	17.05.2022	17	29.10.20	7	29.10.20	24	11.04.22	0.58
28.04.22	67300.00	17.05.2022	5.60	26.04.2022	1.02	12.05.2022	3.52	12.05.2022	15					02.03.22	0.64
26.04.22	50900.00	12.05.2022	10.26	21.04.2022	0.84	10.05.2022	4.20	10.05.2022	<15					14.09.21	0.65
25.04.22	117000.00	10.05.2022	7.24	20.04.2022	0.74	07.05.2022	4.45	07.05.2022	<15						
18.04.22	26800.00	07.05.2022	8.40	02.03.2022	1.41	06.05.2022	3.76	06.05.2022	<15						
15.04.22	46400.00	06.05.2022	9.36	05.08.2020	1.23	03.05.2022	5.39	03.05.2022	<15						
14.04.22	12400.00	03.05.2022	9.20			02.05.2022	4.56	02.05.2022	<15						
02.03.22	2260.00	02.05.2022	10.17			01.05.2022	3.35	01.05.2022	16						
14.09.21	<2000	01.05.2022	9.51			30.04.2022	4.16	30.04.2022	<15						
22.03.21	<2000	30.04.2022	9.49			29.04.2022	3.48	29.04.2022	16						
		29.04.2022	11.69			28.04.2022	4.15	28.04.2022	<15						
		28.04.2022	8.25			26.04.2022	3.13	26.04.2022	18						
		26.04.2022	6.53			26.04.2022	2.24	26.04.2022	27						
		26.04.2022	5.89			25.04.2022	2.96	25.04.2022	19						
		25.04.2022	7.08			24.04.2022	2.07	24.04.2022	29						
		24.04.2022	8.97			23.04.2022	2.86	23.04.2022	20						
		23.04.2022	8.99			22.04.2022	2.15	22.04.2022	28						
		22.04.2022	4.87			21.04.2022	2.92	21.04.2022	19						
		21.04.2022	4.86			20.04.2022	1.91	20.04.2022	32						
		20.04.2022	4.79			19.04.2022	3.13	19.04.2022	18						
		19.04.2022	4.41			18.04.2022	2.27	18.04.2022	26						
		18.04.2022	3.93			17.04.2022	3.07	17.04.2022	18						

		17.04.2022	5.59			16.04.2022	2.21	16.04.2022	27						
		16.04.2022	4.17			15.04.2022	2.84	15.04.2022	20						
		15.04.2022	5.71			14.04.2022	2.07	14.04.2022	29						
		14.04.2022	5.89			13.04.2022	1.62	13.04.2022	39						
		13.04.2022	7.68			12.04.2022	3.65	12.04.2022	<15						
		12.04.2022	6.88			12.04.2022	3.49	12.04.2022	16						
		12.04.2022	6.14			11.04.2022	4.24	11.04.2022	<15						
		11.04.2022	8.23			02.03.2022	2.91	02.03.2022	20						
		02.03.2022	8.00			01.12.2021	2.80	01.12.2021	21						
		01.12.2021	6.95			14.09.2021	2.88	14.09.2021	20						
		14.09.2021	6.45			14.06.2021	2.94	14.06.2021	19						
		14.06.2021	7.42			22.03.2021	2.87	22.03.2021	20						
		22.03.2021	9.08												

Case 6

CMV-DNA	cop/ml	Leukocytes	/nl	Lymphocytes	/nl	Kreatinin	mg/dl	eGFR	ml/min	ALT	U/l	GGT	U/l	Bilirubin	mg/dl
07.03.2023	negativ	30.01.2023	7.47	09.01.2023	2.38	30.01.2023	1.77	30.01.2023	40	07.03.23	29	07.03.23	48	09.01.23	0.69
30.01.2023	negativ	09.01.2023	7.43	02.08.2022	1.04	09.01.2023	1.94	09.01.2023	36	03.11.22	26	03.11.22	70	23.06.22	0.29
09.01.2023	3790	30.11.2022	6.85	23.06.2022	2.10	30.11.2022	1.86	30.11.2022	37	06.09.22	42	06.09.22	51	13.06.22	0.23
30.11.2022	<2000	02.08.2022	5.51	17.05.2022	0.80	02.08.2022	1.67	02.08.2022	43					11.06.22	0.29
03.11.2022	<2000	19.07.2022	7.61	09.05.2022	0.81	19.07.2022	1.81	19.07.2022	39					08.06.22	0.25
02.08.2022	<2000	14.07.2022	6.76			14.07.2022	2.21	14.07.2022	30					07.06.22	0.22
19.07.2022	<2000	05.07.2022	6.70			05.07.2022	2.04	05.07.2022	33					03.06.22	0.17
14.07.2022	<2000	28.06.2022	6.60			28.06.2022	1.94	28.06.2022	36					30.05.22	0.26
05.07.2022	13000	27.06.2022	7.35			27.06.2022	1.71	27.06.2022	41					09.05.22	0.47
23.06.2022	51700	24.06.2022	7.47			24.06.2022	1.99	24.06.2022	34						

21.06.2022	89400	23.06.2022	7.10			23.06.2022	1.92	23.06.2022	36						
13.06.2022	28500	21.06.2022	7.54			21.06.2022	1.85	21.06.2022	38						
25.05.2022	<2000	11.06.2022	5.82			13.06.2022	1.68	13.06.2022	42						
17.05.2022	<2000	09.06.2022	4.65			11.06.2022	1.69	11.06.2022	42						
09.05.2022	negativ	08.06.2022	4.68			09.06.2022	1.70	09.06.2022	42						
		07.06.2022	5.35			08.06.2022	1.61	08.06.2022	45						
		03.06.2022	7.06			07.06.2022	1.83	07.06.2022	38						
		01.06.2022	5.76			05.06.2022	1.86	05.06.2022	37						
		31.05.2022	6.76			03.06.2022	1.82	03.06.2022	38						
		30.05.2022	5.28			01.06.2022	1.78	01.06.2022	39						
		27.05.2022	4.73			31.05.2022	1.92	31.05.2022	36						
		25.05.2022	7.96			30.05.2022	1.73	30.05.2022	41						
		24.05.2022	5.29			27.05.2022	2.15	27.05.2022	31						
		23.05.2022	4.66			25.05.2022	2.21	25.05.2022	30						
		22.05.2022	4.46			24.05.2022	2.37	24.05.2022	28						
		21.05.2022	6.61			23.05.2022	2.48	23.05.2022	26						
		20.05.2022	5.02			22.05.2022	2.27	22.05.2022	29						
		19.05.2022	5.63			21.05.2022	2.05	21.05.2022	33						
		17.05.2022	5.18			20.05.2022	1.88	20.05.2022	37						
		11.05.2022	5.39			19.05.2022	2.02	19.05.2022	34						
		09.05.2022	5.54			17.05.2022	2.05	17.05.2022	33						
						11.05.2022	1.72	11.05.2022	41						
						09.05.2022	1.75	09.05.2022	40						

Case 7

CMV-DNA	cop/ml	Leukocytes	/nl	Lymphocytes	/nl	Kreatinin	mg/dl	eGFR	ml/min	ALT	U/l	GGT	U/l	Bilirubin	mg/dl

02.11.2022	negativ	02.11.2022	5.50	31.10.2022	2.35	02.11.2022	8.83	02.11.2022	<15	24.11.23	22	25.11.23	22	25.11.23	0.20
30.09.2019	2690	31.10.2022	7.18	09.03.2022	1.83	31.10.2022	9.03	31.10.2022	<15	09.03.22	12	09.03.22	15	31.10.22	0.23
24.09.2019	11600	21.03.2022	7.26	05.03.2022	0.90	21.03.2022	3.45	21.03.2022	<15	05.03.22	13	04.03.22	22	21.03.22	0.30
18.09.2019	138000	14.03.2022	6.93	07.09.2020	2.58	14.03.2022	9.42	14.03.2022	<15	04.03.22	14	14.09.20	14	09.03.22	0.28
16.09.2019	78600	09.03.2022	5.72	07.10.2019	0.70	09.03.2022	7.38	09.03.2022	<15	14.09.20	15	09.09.20	17	08.03.22	0.25
13.09.2019	463000	08.03.2022	6.93	04.10.2019	0.55	08.03.2022	5.45	08.03.2022	<15	07.09.20	23	07.09.20	19	07.03.22	0.28
11.09.2019	1260000	07.03.2022	9.61	18.09.2019	0.67	07.03.2022	9.55	07.03.2022	<15	28.09.19	5	28.09.19	18	06.03.22	0.28
04.09.2019	3000000	06.03.2022	13.34	16.09.2019	0.96	06.03.2022	8.18	06.03.2022	<15	28.07.19	8	28.07.19	26	05.03.22	0.30
26.08.2019	368000	05.03.2022	14.91	13.09.2019	1.01	05.03.2022	5.64	05.03.2022	<15	24.07.19	9	24.07.19	26	04.03.22	0.27
19.08.2019	147000	04.03.2022	15.54	13.09.2019	1.01	04.03.2022	4.13	04.03.2022	<15	15.07.19	12	15.07.19	20	14.09.20	0.24
05.08.2019	69200	14.09.2020	5.14	06.09.2019	1.30	14.09.2020	7.54	14.09.2020	<15	13.07.19	10	13.07.19	20	07.09.20	0.20
01.08.2019	87900	13.09.2020	4.71	04.09.2019	1.30	10.09.2020	4.81	10.09.2020	<15	11.07.19	8	11.07.19	16	23.09.19	0.25
22.07.2019	1920000	10.09.2020	5.80	02.08.2019	0.98	09.09.2020	7.23	09.09.2020	<15	09.07.19	8	09.07.19	16	18.09.19	0.24
17.07.2019	2620000	09.09.2020	5.78	31.07.2019	0.66	07.09.2020	8.06	07.09.2020	<15	05.07.19	15	05.07.19	17	11.09.19	0.16
12.07.2019	1620000	07.09.2020	6.58	28.07.2019	0.30	05.10.2019	3.90	05.10.2019	<15	01.07.19	18	01.07.19	24	06.09.19	0.25
08.07.2019	563000	07.10.2019	4.66	26.07.2019	0.99	05.10.2019	3.90	05.10.2019	<15	29.06.19	15	29.06.19	22	04.09.19	0.20
05.07.2019	111000	05.10.2019	4.25	24.07.2019	0.65	04.10.2019	5.95	04.10.2019	<15	28.06.19	22	28.06.19	23	26.08.19	0.25
29.06.2019	21700	05.10.2019	4.25	22.07.2019	0.52	02.10.2019	6.56	02.10.2019	<15	28.06.19	19	28.06.19	21	23.08.19	0.21
25.06.2019	25500	04.10.2019	4.51	21.07.2019	0.82	01.10.2019	5.05	01.10.2019	<15	25.06.19	10	25.06.19	21	28.07.19	0.32
17.06.2019	17400	02.10.2019	4.76	11.07.2019	0.37	30.09.2019	5.96	30.09.2019	<15	25.06.19	11	25.06.19	20	21.07.19	0.31
05.06.2019	11900	01.10.2019	5.44	10.07.2019	0.42	28.09.2019	5.71	28.09.2019	<15	17.06.19	11	17.06.19	19	15.07.19	0.21
23.05.2019	12100	30.09.2019	6.63	05.06.2019	0.48	24.09.2019	5.25	24.09.2019	<15	05.06.19	14	05.06.19	20	09.07.19	0.25
09.05.2019	16800	28.09.2019	7.57	25.04.2019	0.52	23.09.2019	7.31	23.09.2019	<15	23.05.19	21	23.05.19	27	05.07.19	0.34
25.04.2019	<2000	25.09.2019	8.67	09.04.2019	1.33	18.09.2019	1.53	18.09.2019	38	09.05.19	22	09.05.19	21	29.06.19	0.27
07.04.2019	<2000	24.09.2019	9.41	26.02.2019	0.31	16.09.2019	4.55	16.09.2019	<15	25.04.19	15	25.04.19	22	28.06.19	0.28
04.04.2019	<2000	23.09.2019	8.99			13.09.2019	3.87	13.09.2019	<15	15.04.19	13	15.04.19	27	25.06.19	0.23
27.02.2019	negativ	20.09.2019	3.33			13.09.2019	3.87	13.09.2019	<15	09.04.19	11	04.04.19	17	09.04.19	0.35
		18.09.2019	3.65			11.09.2019	1.55	11.09.2019	38	04.04.19	7	03.04.19	22	04.04.19	0.36

16.09.2019	4.84		04.09.2019	4.39	04.09.2019	<15	03.04.19	9	15.03.19	28	15.03.19	0.16
13.09.2019	4.44		02.09.2019	4.73	02.09.2019	<15	15.03.19	9	15.03.19	25	13.03.19	0.22
13.09.2019	4.44		02.09.2019	4.73	02.09.2019	<15	15.03.19	12	13.03.19	23	28.02.19	0.17
11.09.2019	3.92		30.08.2019	1.53	30.08.2019	38	13.03.19	9	28.02.19	23		
09.09.2019	4.20		26.08.2019	4.87	26.08.2019	<15	28.02.19	13	26.02.19	32		
06.09.2019	5.95		23.08.2019	4.58	23.08.2019	<15	26.02.19	14				
04.09.2019	5.18		02.08.2019	5.66	02.08.2019	<15						
26.08.2019	5.70		31.07.2019	5.20	31.07.2019	<15						
23.08.2019	6.63		29.07.2019	6.28	29.07.2019	<15						
16.08.2019	8.79		28.07.2019	5.56	28.07.2019	<15						
12.08.2019	7.39		26.07.2019	5.82	26.07.2019	<15						
09.08.2019	6.57		24.07.2019	6.40	24.07.2019	<15						
02.08.2019	8.81		21.07.2019	6.49	21.07.2019	<15						
01.08.2019	7.58		19.07.2019	7.07	19.07.2019	<15						
31.07.2019	7.70		17.07.2019	7.48	17.07.2019	<15						
29.07.2019	7.43		15.07.2019	8.12	15.07.2019	<15						
28.07.2019	5.28		13.07.2019	4.80	13.07.2019	<15						
26.07.2019	7.80		12.07.2019	6.91	12.07.2019	<15						
24.07.2019	5.56		11.07.2019	8.10	11.07.2019	<15						
22.07.2019	4.19		10.07.2019	6.79	10.07.2019	<15						
21.07.2019	4.89		09.07.2019	4.80	09.07.2019	<15						
19.07.2019	3.39		08.07.2019	3.38	08.07.2019	<15						
17.07.2019	4.32		07.07.2019	2.31	07.07.2019	23						
15.07.2019	5.32		06.07.2019	1.82	06.07.2019	31						
13.07.2019	3.77		05.07.2019	1.61	05.07.2019	36						
12.07.2019	4.30		01.07.2019	1.47	01.07.2019	40						
11.07.2019	3.76		29.06.2019	1.65	29.06.2019	35						
10.07.2019	3.20		28.06.2019	1.94	28.06.2019	29						

09.07.2019	2.49		28.06.2019	1.88	28.06.2019	30		
08.07.2019	2.00		25.06.2019	2.37	25.06.2019	23		
07.07.2019	2.32		25.06.2019	2.39	25.06.2019	22		
06.07.2019	2.45		17.06.2019	1.65	17.06.2019	35		
05.07.2019	2.66		05.06.2019	1.64	05.06.2019	35		
01.07.2019	3.00		23.05.2019	2.12	23.05.2019	26		
29.06.2019	2.72		09.05.2019	2.49	09.05.2019	21		
28.06.2019	3.17		25.04.2019	2.50	25.04.2019	21		
28.06.2019	3.13		15.04.2019	3.53	15.04.2019	<15		
25.06.2019	3.75		11.04.2019	3.62	11.04.2019	<15		
25.06.2019	3.92		10.04.2019	3.65	10.04.2019	<15		
17.06.2019	4.06		09.04.2019	3.88	09.04.2019	<15		
05.06.2019	4.59		07.04.2019	3.88	07.04.2019	<15		
23.05.2019	3.77		05.04.2019	4.18	05.04.2019	<15		
09.05.2019	4.74		04.04.2019	4.71	04.04.2019	<15		
25.04.2019	2.73		03.04.2019	4.60	03.04.2019	<15		
15.04.2019	5.22		15.03.2019	4.21	15.03.2019	<15		
11.04.2019	4.62		15.03.2019	4.10	15.03.2019	<15		
10.04.2019	4.39		13.03.2019	3.88	13.03.2019	<15		
09.04.2019	4.30		07.03.2019	2.59	07.03.2019	20		
07.04.2019	5.01		05.03.2019	2.90	05.03.2019	18		
05.04.2019	5.67		04.03.2019	2.95	04.03.2019	17		
04.04.2019	4.61		02.03.2019	2.82	02.03.2019	18		
03.04.2019	5.57		28.02.2019	3.39	28.02.2019	<15		
15.03.2019	5.42		27.02.2019	3.30	27.02.2019	15		
15.03.2019	5.52							
13.03.2019	5.90							
07.03.2019	4.32							

		05.03.2019	3.01												
		04.03.2019	2.29												
		02.03.2019	2.81												
		01.03.2019	3.52												
		28.02.2019	4.09												
		27.02.2019	4.58												

Case 8

CMV-DNA	cop/ml	Leukocytes	/nl	Lymphocytes	/nl	Kreatinin	mg/dl	eGFR	ml/min	ALT	U/l	GGT	U/l	Bilirubin	mg/dl
08.02.2023	negativ	08.02.2023	13.27	08.02.2023	3.12	08.02.2023	1.54	08.02.2023	37	08.02.23	37	08.02.23	89	08.02.23	0.30
08.04.2022	<2000	26.10.2022	12.63	08.04.2022	3.49	26.10.2022	1.54	26.10.2022	37	26.10.22	29	26.10.22	75	08.04.22	0.22
21.03.2022	negativ	27.07.2022	13.86	28.03.2022	3.58	27.07.2022	1.49	27.07.2022	39	08.04.22	15	08.04.22	156	21.03.22	0.35
25.02.2022	<2000	08.04.2022	16.53	25.03.2022	2.95	08.04.2022	2.00	08.04.2022	27	26.01.22	40	26.01.22	186	25.02.22	0.37
26.01.2022	negativ	28.03.2022	10.19	21.03.2022	1.61	28.03.2022	1.49	28.03.2022	39	03.11.21	38	03.11.21	155	26.01.22	0.16
03.11.2021	negativ	25.03.2022	9.93	25.02.2022	2.00	25.03.2022	1.49	25.03.2022	39	08.09.21	33	08.09.21	108	03.11.21	0.19
08.09.2021	negativ	23.03.2022	9.77	26.01.2022	2.25	23.03.2022	1.60	23.03.2022	36	30.06.21	29	30.06.21	134	08.09.21	0.18
26.03.2021	negativ	22.03.2022	11.96	03.11.2021	1.65	22.03.2022	1.84	22.03.2022	30	26.03.21	31	26.03.21	94	26.03.21	0.17
28.12.2020	negativ	21.03.2022	11.75	08.09.2021	1.41	21.03.2022	1.98	21.03.2022	28	28.12.20	22	23.12.20	178	28.12.20	<0.15
22.12.2020	negativ	07.03.2022	9.50	26.03.2021	1.08	07.03.2022	1.48	07.03.2022	40	25.12.20	25	22.12.20	206	25.12.20	0.20
23.09.2020	<2000	03.03.2022	8.58	25.12.2020	1.15	04.03.2022	1.40	04.03.2022	42	23.12.20	29	23.09.20	82	23.12.20	0.19
23.09.2020	<2000	02.03.2022	13.36	22.12.2020	0.38	03.03.2022	1.29	03.03.2022	47	22.12.20	37	26.08.20	67	22.12.20	0.20
26.08.2020	<2000	01.03.2022	7.92	23.09.2020	0.67	02.03.2022	1.25	02.03.2022	49	26.11.20	9			23.09.20	0.24
15.07.2020	51400	28.02.2022	9.84	15.07.2020	1.66	01.03.2022	1.21	01.03.2022	50	23.11.20	20			15.07.20	0.19
17.06.2020	29100	25.02.2022	8.99	17.06.2020	1.26	28.02.2022	1.32	28.02.2022	45	23.09.20	25				
10.06.2020	62300	25.02.2022	7.43	26.05.2020	1.29	25.02.2022	1.78	25.02.2022	32	26.08.20	12				
26.05.2020	23000	24.02.2022	11.70	18.04.2020	1.77	25.02.2022	1.75	25.02.2022	32						

13.05.2020	32400	09.02.2022	7.80	15.04.2020	0.69	24.02.2022	1.77	24.02.2022	32
16.04.2020	596000	08.02.2022	9.82	03.03.2020	0.33	09.02.2022	1.50	09.02.2022	39
08.04.2020	2180000	07.02.2022	11.29	24.01.2020	0.05	08.02.2022	1.55	08.02.2022	37
03.03.2020	26800	05.02.2022	10.95	15.01.2020	0.09	07.02.2022	1.66	07.02.2022	34
24.01.2020	15200	05.02.2022	9.77	23.09.2019	0.19	05.02.2022	1.50	05.02.2022	39
15.01.2020	11000	03.02.2022	12.73	26.06.2019	0.17	05.02.2022	1.43	05.02.2022	41
20.11.2019	2070	26.01.2022	11.32	26.03.2019	offen	03.02.2022	1.37	03.02.2022	43
26.06.2019	negativ	03.11.2021	12.12	22.03.2019	0.16	26.01.2022	1.60	26.01.2022	36
02.05.2019	<2000	08.09.2021	8.26	15.03.2019	0.06	03.11.2021	1.59	03.11.2021	36
08.04.2019	<2000	30.06.2021	8.35	08.01.2019	0.15	08.09.2021	1.68	08.09.2021	34
01.04.2019	8310	26.03.2021	9.30	04.12.2018	0.22	30.06.2021	1.58	30.06.2021	37
29.03.2019	57300	24.12.2020	7.81	06.11.2018	0.31	26.03.2021	2.07	26.03.2021	27
25.03.2019	230000	25.11.2020	8.70	09.10.2018	0.20	28.12.2020	0.96	28.12.2020	67
22.03.2019	122000	23.09.2020	7.59	25.09.2018	0.24	27.12.2020	1.01	27.12.2020	63
20.03.2019	474000	26.08.2020	8.13	18.09.2018	0.08	25.12.2020	1.19	25.12.2020	52
18.03.2019	3680000	15.07.2020	8.51	13.09.2018	0.08	25.12.2020	1.20	25.12.2020	51
15.03.2019	1940000	17.06.2020	9.56	03.09.2018	0.17	24.12.2020	1.22	24.12.2020	50
05.02.2019	<2000	26.05.2020	8.71	02.09.2018	0.13	23.12.2020	1.28	23.12.2020	48
08.01.2019	negativ	13.05.2020	8.04	01.09.2018	0.07	22.12.2020	1.10	22.12.2020	57
04.12.2018	negativ	08.04.2020	6.95	31.08.2018	0.28	30.11.2020	1.90	30.11.2020	29
06.11.2018	negativ	03.03.2020	10.83	30.08.2018	0.14	26.11.2020	1.93	26.11.2020	29
09.10.2018	34100	24.01.2020	7.52			23.11.2020	1.42	23.11.2020	42
30.08.2018	negativ	20.11.2019	10.14			23.09.2020	1.31	23.09.2020	46
		23.09.2019	10.71			26.08.2020	1.50	26.08.2020	39
		26.06.2019	9.96						
		02.05.2019	7.86						
		01.04.2019	2.84						
		23.03.2019	1.13						

14.03.2019	3.27
05.02.2019	10.46
08.01.2019	5.49
04.12.2018	12.33
06.11.2018	10.44
13.09.2018	7.38
30.08.2018	10.63
